# High Extracellular Ca^2+^ Stimulates Ca^2+^-Activated Cl^−^ Currents in Frog Parathyroid Cells through the Mediation of Arachidonic Acid Cascade

**DOI:** 10.1371/journal.pone.0019158

**Published:** 2011-04-29

**Authors:** Yukio Okada, Kotapola G. Imendra, Toshihiro Miyazaki, Hitoshi Hotokezaka, Rie Fujiyama, Kazuo Toda

**Affiliations:** 1 Integrative Sensory Physiology, Graduate School of Biomedical Science, Nagasaki University, Nagasaki, Nagasaki, Japan; 2 Cell Biology, Graduate School of Biomedical Science, Nagasaki University, Nagasaki, Nagasaki, Japan; 3 Orthodontics and Biomedical Engineering, Graduate School of Biomedical Science, Nagasaki University, Nagasaki, Nagasaki, Japan; 4 Department of Physiology, Faculty of Medicine, University of Ruhuna, Galle, Sri Lanka; University of Oldenburg, Germany

## Abstract

Elevation of extracellular Ca^2+^ concentration induces intracellular Ca^2+^ signaling in parathyroid cells. The response is due to stimulation of the phospholipase C/Ca^2+^ pathways, but the direct mechanism responsible for the rise of intracellular Ca^2+^ concentration has remained elusive. Here, we describe the electrophysiological property associated with intracellular Ca^2+^ signaling in frog parathyroid cells and show that Ca^2+^-activated Cl^−^ channels are activated by intracellular Ca^2+^ increase through an inositol 1,4,5-trisphophate (IP_3_)-independent pathway. High extracellular Ca^2+^ induced an outwardly-rectifying conductance in a dose-dependent manner (EC_50_∼6 mM). The conductance was composed of an instantaneous time-independent component and a slowly activating time-dependent component and displayed a deactivating inward tail current. Extracellular Ca^2+^-induced and Ca^2+^ dialysis-induced currents reversed at the equilibrium potential of Cl^−^ and were inhibited by niflumic acid (a specific blocker of Ca^2+^-activated Cl^−^ channel). Gramicidin-perforated whole-cell recording displayed the shift of the reversal potential in extracellular Ca^2+^-induced current, suggesting the change of intracellular Cl^−^ concentration in a few minutes. Extracellular Ca^2+^-induced currents displayed a moderate dependency on guanosine triphosphate (GTP). All blockers for phospholipase C, diacylglycerol (DAG) lipase, monoacylglycerol (MAG) lipase and lipoxygenase inhibited extracellular Ca^2+^-induced current. IP_3_ dialysis failed to induce conductance increase, but 2-arachidonoylglycerol (2-AG), arachidonic acid and 12S-hydroperoxy-5Z,8Z,10E,14Z-eicosatetraenoic acid (12(S)-HPETE) dialysis increased the conductance identical to extracellular Ca^2+^-induced conductance. These results indicate that high extracellular Ca^2+^ raises intracellular Ca^2+^ concentration through the DAG lipase/lipoxygenase pathway, resulting in the activation of Cl^−^ conductance.

## Introduction

Parathyroid hormone (PTH) regulates extracellular free Ca^2+^ concentration ([Ca^2+^]_o_) in cooperation with 1,25-dihydroxycholecalciferol (1,25-(OH)_2_D_3_)and calcitonin. On the other hand, [Ca^2+^]_o_ regulates the secretion of PTH from parathyroid cells through an extracellular Ca^2+^-sensing receptor (CaR) [1], [2]. High [Ca^2+^]_o_ inhibits the secretion, whereas low [Ca^2+^]_o_ enhances the secretion. It is believed that extracellular Ca^2+^ binds to CaR, and as a consequence inhibits the secretion of PTH via intracellular free Ca^2+^ concentration ([Ca^2+^]_i_). However, the molecular mechanism by which [Ca^2+^]_i_ regulates the secretion is not well elucidated.

The CaR belongs to the family C of G protein-coupled receptors (GPCRs) and has a large extracellular domain that binds external Ca^2+^ and other CaR agonists. The CaR controls various signaling pathways [3]–[5]. Calcium binding to the receptor results in G protein-dependent activation of phosphatidylinositol-specific phospholipase C (PI-PLC) causing accumulation of inositol 1,4,5-trisphosphate (IP_3_) and diacylglycerol (DAG) and promoting rapid release of Ca^2+^ from its intracellular stores [6], [7]. The CaR-mediated activation of PI-PLC in parathyroid cells is a direct G protein-mediated process, while activation of phospholipase A_2_ (PLA_2_) and D by high [Ca^2+^]_o_ are probably indirect, through the mediation of PLC-dependent activation of protein kinase C [4].

DAG can be utilized for 2-arachidonoylglycerol (2-AG) generation [8]. PLC hydrolyzes phosphatidylinositol and produces arachidonic acid-containing DAG. Then, DAG is converted into 2-AG by the action of DAG lipase. Next, 2-AG is hydrolyzed by monoacylglycerol (MAG) lipase and yields arachidonic acid. Finally, arachidonic acid is oxidized by cycloxygenase (COX), lipoxygenase (LO) or epoxygenase (cytochrome P450).

The mitogen-activated protein kinase (MAP kinase) pathways are found in bovine parathyroid cells [9]. MAP kinase is activated by dual tyrosine and threonine phosphorylation [10]. Phosphorylated MAP kinase can phosphorylate cytosolic phospholipase A_2_ (cPLA_2_) [11]. In bovine parathyroid cells, the MAP kinase is activated by CaR [9]. There are several mechanisms by which GPCRs stimulate MAP kinase. Gβγ subunits stimulate MAP kinase pathway by activating Src-family tyrosine kinase.

The electrophysiological studies using classical intracellular microelectrodes indicated that rodent parathyroid cells display a deep resting potential (about −70 mV), which is depolarized by increasing [Ca^2+^]_o_ [12], [13]. Later, the patch-clamp technique was applied on bovine, human and rodent parathyroid cells. [14]–[19]. These studies showed that parathyroid cells possess some types of K^+^ channels. Other studies suggested the presence of voltage-gated Ca^2+^ channels in bovine, goat and human parathyroid cells [20]–[22]. However, a recent study claimed that human parathyroid cells lack voltage-gated Ca^2+^ channels, and that TRPC ion channels associated with Orai1 and STIM1 may increase intracellular Ca^2+^ concentration in the cells [23]. Frog parathyroid cells possess voltage-gated Na^+^ channels in contrast to mammalian cells [24].

Increase in [Ca^2+^]_o_ and CaR agonists raise [Ca^2+^]_i_ in bovine parathyroid cells and inhibit PTH secretion [25], [26]. Ion channels are regulated by neurotransmitter and hormones via GPCRs [27], [28]. GPCRs dissociate heterotrimeric G proteins (Gαβγ) to Gα-GTP and Gβγ. Both subunits can regulate a variety of ion channels directly (via physical interactions between G protein subunits and the channel protein) or indirectly (via second messengers and protein kinases). Increase of [Ca^2+^]_i_ activates Ca^2+^-activated K^+^ channels in human parathyroid cell [19].

In the present study, we report that frog parathyroid cells possess Ca^2+^-activated Cl^−^ channels and that these channels can be activated by an increase of [Ca^2+^]_i_ through the mediation of the arachidonic acid cascade.

## Results

### Intracellular Ca^2+^ Increase in Response to Calciminetics and High Extracellular Ca^2+^

**Figure 1:**
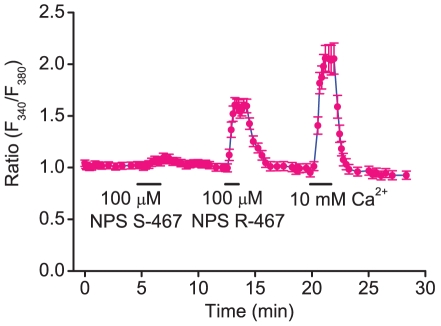
Intracellular Ca^2+^-imaging in isolated frog parathyroid cells. Extracellular application of 100 µM NPS R-467 and 10 mM Ca^2+^ induced large responses in 26 cells, but 100 µM NPS S-467 is less effective than R-467.

Calcimimetics can cause an increase in intracellular Ca^2+^ concentration [Ca^2+^]_i_ in mammalian parathyroid cells [26]. We assessed the effect of calcimimetics on [Ca^2+^]_i_ of frog parathyroid cells. NPS-R-467 and extracellular 10 mM Ca^2+^ saline solution induced a large increase in [Ca^2+^]_i_, but NPS S-467 was less effective than the R-467 (Figure 1). The result indicates that frog parathyroid cells discriminate the stereo-selective difference between the enantiomers. NPS-R467 and 10 mM Ca^2+^ increased [Ca^2+^]_i_ in about 90% of the cells.

### Characteristics of Extracellular Ca^2+^-induced Current in Frog Parathyroid Cells

Basal properties of frog parathyroid cells have been reported in a previous paper [24]. Briefly, under conventional whole-cell mode in a standard K^+^ internal solution, frog parathyroid cells displayed a resting potential of about −30 mV, input resistance of 13 GΩ and membrane capacitance of 8 pF. There was no difference between conventional and perforated conditions in those properties. After attaining the perforated whole-cell configuration in normal saline solution, the whole-cell current-voltage (*I/V*) relationships produced by a voltage ramp (167 mV/s) from −100 to +100 mV were almost linear in normal saline solution (data not shown).

**Figure 2:**
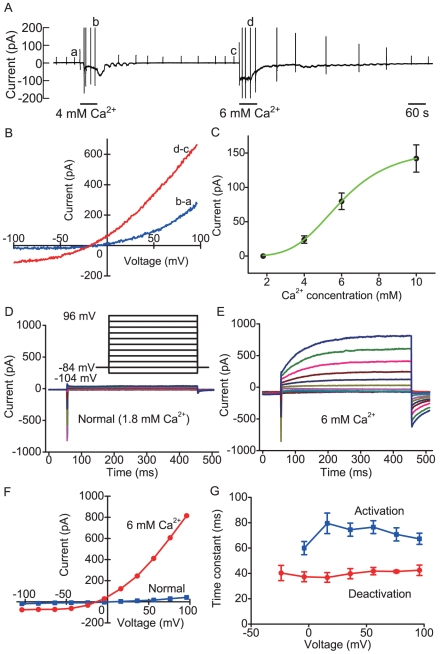
Extracellular Ca^2+^-induced current in frog parathyroid cells. (A)The pen recording of the effect of high extracellular Ca^2+^ on the current signal measured at a holding potential of −54 mV. The transient outward current deflections larger than 200 pA in the pen recording are out of scale. (B) The plots of the whole-cell current/voltage (*I/V*) relationships produced by a voltage ramp (167 mV/s) from −100 to +100 mV. *I/V* relationships labeled “b-a” and “d-c” are the differences between a and b in pen recording and that between c and d in the recording, respectively. The same description mode was applied in the following figures. (C) Dose-response relationship for extracellular Ca^2+^-induced current measured at −54 mV. The ordinate scale denotes the absolute value of the inward current. Data were fitted by the Hill equation (Hill coefficient: 4.3). Values are mean ± SEM from 10–16 cells. (D)The currents elicited by 400 ms voltage steps between −104 to +96 mV in 20 mV increments from a holding potential of −84 mV in normal saline solution. (E) The currents in extracellular 6 mM Ca^2+^ saline solution. The initial transient inward currents are the voltage-gated Na^+^ currents. The leak currents were not subtracted from the current traces. (F) Current-voltage (*I/V*) relationships for the currents measured at the end of the pulse in both conditions. (G) The time constants of activation and deactivation of extracellular Ca^2+^-induced currents as function of membrane potential. Values are mean ± SEM from 4 cells. In this figure, the pipette solution contained amphotericin B (133 µg/ml).

**Figure 3:**
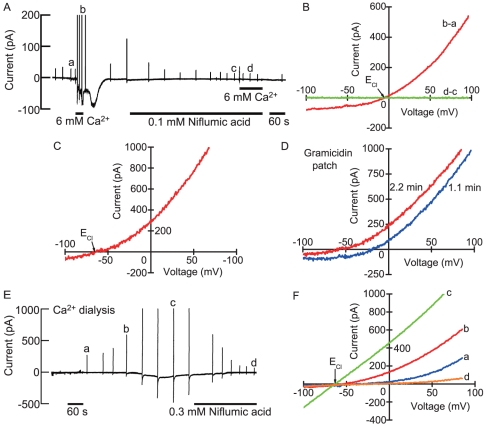
The dependence of the reversal potentials of extracellular Ca^2+^-induced and Ca^2+^ dialysis-induced currents on intracellular Cl^−^ concentration. (A)The pen recording of the inhibitory effect of niflumic acid on extracellular Ca^2+^-induced current measured at a holding potential of −54 mV. (B) The plot of the whole-cell current/voltage (*I/V*) relationships produced by a voltage ramp of the extracellular Ca^2+^-induced currents obtained from the pen recording on the upper. (C) The shift of the reversal potential of extracellular Ca^2+^-induced current in another cell. Intracellular Cl^−^ concentration was reduced from 104 to 10 mM. In Figure 3A, B and C, the pipette solution contained amphotericin B (133 µg/ml). (D)The gramicidin-perforated whole-cell current/voltage (*I/V*) relationships by a voltage ramp of the extracellular Ca^2+^-induced currents. Only in this case, the pipette solution contained gramicidin (100 µg/ml). (E) The pen recording of intracellular 0.8 mM Ca^2+^-induced current and its inhibition with niflumic acid. (F) The plots of *I/V* relationships produced by a voltage ramp of Ca^2+^ dialysis-induced currents. *I/V* relationships labeled a–d were obtained at the time indicated by the same letters on the pen recording.

When the extracellular Ca^2+^ concentration was increased from 1.8 mM to a higher concentration, frog parathyroid cells displayed a sustained inward current at a holding potential of −54 mV (Figure 2A). *I/V* relationships produced by a voltage ramp indicated an outwardly rectifying property in high Ca^2+^ saline solution (Figure 2B). However, at 10 mM Ca^2+^, linear *I/V* relationships were observed in 5 of 10 cells. The magnitude of the current increased in a dose-dependent manner of Ca^2+^ (Figure 2C). The apparent EC_50_ for extracellular Ca^2+^ was about 6 mM. The biophysical properties of extracellular Ca^2+^-induced currents elicited by voltage steps between −104 mV and +96 mV for 400 ms from a holding potential of −84 mV were examined. Frog parathyroid cells displayed transient inward currents and subsequent sustained leak currents in response to depolarizing steps in normal saline solution (Figure 2D). We have reported previously that the transient inward currents were the tetrodotoxin (TTX)-sensitive, voltage-gated Na^+^ currents [24]. When extracellular Ca^2+^ was increased to 6 mM, slowly developing but sustained outward currents appeared in response to depolarizing steps (Figure 2E). Deactivating inward tail currents were also observed on the return to −84 mV from depolarizing potentials (Figure 2E). Outward currents were composed of an instantaneous time-independent component and a slowly activating time-dependent component. The steady-state *I/V* relationships measured at the end of the pulse changed from almost linear characteristic (normal saline) to outwardly rectifying (6 mM Ca^2+^) (Figure 2F).The activation and deactivation time constants did not show clear dependence on the voltage (Figure 2G). Extracellular 6 mM Ca^2+^ shifted the threshold of the voltage for the activation of transient inward Na^+^ currents from −54 mV to −44 mV reversibly. This shift may be due to the change of surface charge elicited by high Ca^2+^. The biophysical properties of extracellular Ca^2+^-induced currents suggested the appearance of Ca^2+^-activated Cl^−^ current [29]. Niflumic acid (0.1 mM, a specific blocker of Ca^2+^-activated Cl^−^ channel) inhibited extracellular Ca^2+^-induced currents completely (n = 3)(Figure 3A and B). The currents had the reversal potential of −14±3 mV (n = 7) close to the E_Cl_ (−7 mV). When intracellular Cl^−^ concentration decreased from 104 to 10 mM, the reversal potential shifted to −69±3 mM (n = 5) when E_Cl_ was set to −66 mV (Figure 3C). Prevention of the permeation of Cl^−^ through the patch membrane using gramicidin instead of amphotericin B caused the reversal potential of extracellular Ca^2+^-induced current to change from −25±2 mV in early stage (1 min) to −58±3 mV in steady state (3 min) (n = 4)(Figure 3D). When the pipette was filled with a modified Cs^+^, low Cl^−^ (10 mM) internal solution (E_Cl_ = −63 mV) containing 0.8 mM Ca^2+^ without EGTA, frog parathyroid cells displayed a gradual increase in outward-rectifying current (Figure 3E and F). The outward-rectifying current was strongly inhibited by niflumic acid (Figure 3E) and the niflumic acid-sensitive current had a mean reversal potential of −63±2 mV (n = 5) (Figure 3F).

Nominal Ca^2+^-free 10 mM Ba^2+^ solution without Na^+^ also induced a conductance increase and the currents had the reversal potential close to E_Cl_ (Figure S1A and B). Even though extracellular Ca^2+^ was eliminated by 1 mM EGTA, a Ca^2+^-deficient 10 mM Ba^2+^ solution could induce the outwardly-rectifying current as well as the Ba^2+^ solution containing 1.8 mM Ca^2+^ did (Figure S1C, D and E). Extracellular 6 mM Sr^2+^ displayed about half of the potency of 6 mM Ca^2+^. Extracellular 0.1 mM Gd^3+^ did not induce any response in frog parathyroid cells, while the drug inhibited extracellular Ca^2+^-induced current strongly, although a previous study has reported that Ga^3+^ induced a large response in *Xenopus* oocytes injected with cRNA for bovine Ca^2+^-sensing receptor [1]. Spermine (1 mM) also did not induce any response in frog parathyroid cells. NPS R-467 (0.1 mM) induced the outwardly rectifying currents in 2 of 4 cells when E_Cl_ was set to −63 mV, but other R-467-insensitive cells displayed a moderate response to extracellular 10 mM Ca^2+^.

### Effects of Intracellular Drugs Related to Phospholipase C on Extracellular Ca^2+^-induced Current

**Figure 4:**
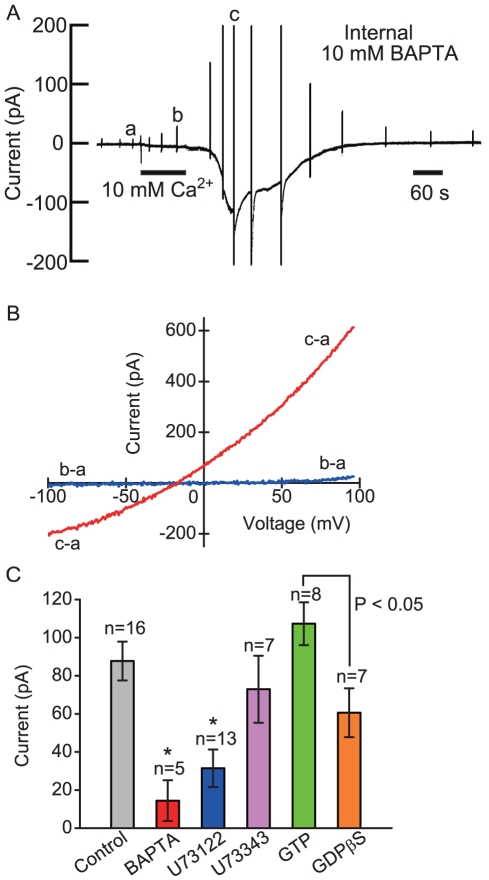
Effects of intracellular drugs on extracellular Ca^2+^-induced current. (A)The pen recording of the inhibitory effect of internal 10 mM BAPTA on extracellular Ca^2+^-induced current measured at −54 mV. (B) The plot of the whole-cell current/voltage (*I/V*) relationships produced by a voltage ramp. (C) Comparison of the effects of intracellular drugs on extracellular 10 mM Ca^2+^-induced current measured at −54 mV.

**Figure 5:**
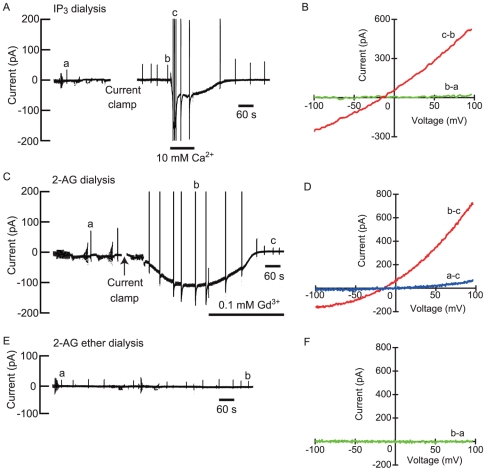
Effects of intracellular 1,4,5-IP_3_, 2-AG and 2-AG ether. (A),(C),(E) The pen recordings of the current signals measured at −54 mV during 50 µM IP_3_ dialysis, 50 µM 2-AG dialysis and 50 µM 2-AG ether dialysis. (B),(D),(F) The plots of the whole-cell current/voltage (*I/V*) relationships produced by a voltage ramp. *I/V* relationships were obtained from the pen recordings on the left. The breaks in the pen recordings denote the condition of current clamp mode.

After attaining the conventional whole-cell configuration with a standard K^+^ internal solution without GTP, frog parathyroid cells still displayed a sustained inward current of −88±10 pA (n = 16) at −54 mV in response to extracellular 10 mM Ca^2+^, although repetitive recordings were impossible. In the conventional mode, the effects of drugs were estimated by the different responses of different populations of cells. When the internal Ca^2+^-buffer of 1 mM EGTA was replaced with 10 mM BAPTA, the extracellular 10 mM Ca^2+^-induced current decreased to 16% of the controls (Figure 4). During washout, a slowly developing but transient conductance increase always appeared (Figure 4A and B). In the present study, further analysis of the response was avoided. Addition of 10 µM U73122 (an inhibitor of phopholipase C) into the internal solution decreased the current response to 36% of the controls, which was a significant decreases, but U73343 (a weak analog of U73122) did not affect the current response. Addition of 1 mM GTP and 1 mM GDPβS did not change the magnitude of the current significantly. However, there was a significant difference in current magnitudes between GTP and GDPβS. Thus, extracellular Ca^2+^-induced current had a moderate dependency on GTP (Figure 4C). Even when the pipette contained 50 µM inositol 1,4,5-trisphosphate, frog parathyroid cells did not display any response, but the cells showed an inward current of −103±20 pA (n = 7) at −54 mV in response to extracellular 10 mM Ca^2+^ (Figure 5A and B). When 50 µM 2-arachidonoylglycerol (2-AG) was dialyzed into the cells, the drug induced a slowly developing inward current of −205±59 pA at −54 mV in 3 of 4 cells within an hour after dissolving the drug in the pipette solution (Figure 5C and D), but the cells (6 cells) did not respond after an hour from dissolving the drug in the pipette solution. Internal 2-arachidonoyglycerol ether (2-AG ether, a chemically stable analog of 2-AG) did not induce any response in the cells (n = 6, Figure 5E and F). Although 2-AG is an endogenous agonist of the cannabinoid-1 (CB_1_) receptor, external 2-AG ether (50 µM) and Win55,212-2 (10 µM, cannabinoid receptor agonist) did not induce a conductance increase. Intracellular 1-oleoyl-2-acethyl-*sn*-glycerol (OAG, 50 µM, a synthetic analog of DAG), cyclic AMP (1 mM) and m-3M3FBS (50 µM, an activator of phospholipase C) also did not elicit any response. Intracellular 20 or 50 µM RHC-80267 (an inhibitor of DAG lipase) did not inhibit extracellular Ca^2+^-induced current, but the conductance increase did not return to basal level after washout, suggesting that the drug has an unknown non-specific effect. Intracellular ryanodine (100 µM) and ruthenium red (30 µM) also did not inhibit extracellular Ca^2+^-induced current.

### Effects of Extracellular Drugs Related to Arachidonic Acid Cascade on Extracellular Ca^2+^-induced current

**Figure 6:**
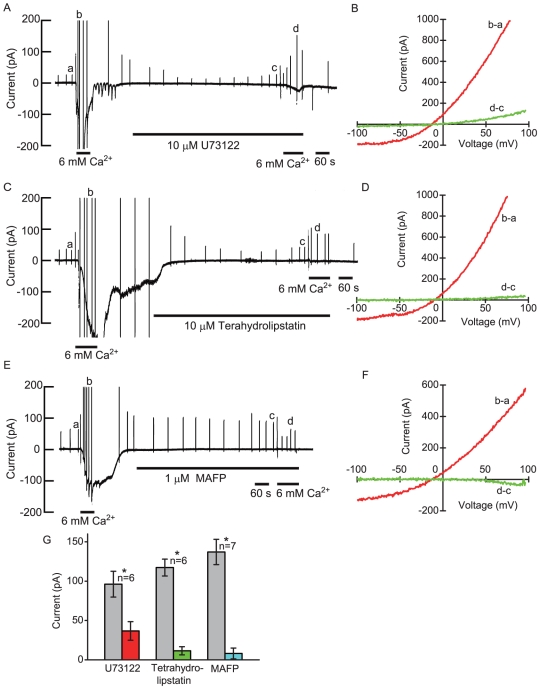
Effects of lipase inhibitors on extracellular Ca^2+^-induced current. (A),(C), (E)The pen recordings of the inhibitory effects of U73122, tetrahydrolipstatin and MAFP on extracellular Ca^2+^-induced current at −54 mV. (B),(D),(F) The plots of the whole-cell *I/V* relationships during control and application of lipase inhibitor. The relationships were obtained from the pen recording on the left. (G) Comparison of the effects of external lipase inhibitors on extracellular Ca^2+^-induced current at −54 mV. The pipette solution contained amphotericin B (133 µg/ml).

**Figure 7:**
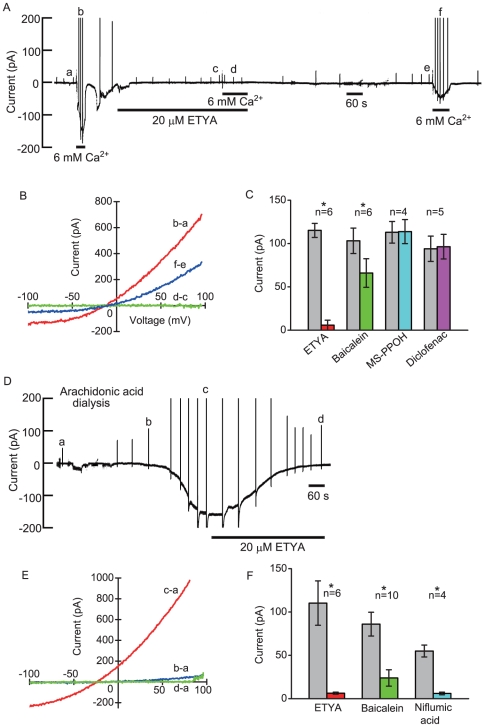
Effects of arachidonic acid-related drugs on the current signals. (A)The pen recording of the inhibitory effect of ETYA on extracellular Ca^2+^-induced current at −54 mV. (B) The plots of the whole-cell *I/V* relationships during control, ETYA application and wash-out. The relationships were obtained from the pen recording on the upper. (C) Comparison of the effects of external 20 µM ETYA, 20 µM baicalein, 20 µM MS-PPOH and 20 µM dicrofenac on extracellular Ca^2+^-induced current at −54 mV. In Figure 7A, B and C, the pipette solution contained amphotericin B (133 µg/ml). (D) The pen recording of the current signal measured at −54 mV during 50 µM arachidonic acid dialysis. (E) The plots of the whole-cell *I/V* relationships during arachidonic acid dialysis. *I/V* relationships were obtained from the pen recording on the upper. (F) Comparison of effects of external 20 µM ETYA, 20 µM baicalein and 100 µM niflumic acid on intracellular arachidonic acid-induced current at −54 mV.

We also checked the effect of external U73122 on the extracellular Ca^2+^-induced current. External administration of the drug decreased the extracellular 6 mM Ca^2+^-induced current to 38% of the controls (Figure 6A and G), which was consistent with the internal effect of U73122 (36%). Although neither IP_3_ nor the DAG analog elicited any response in the phospholipase C pathway of frog parathyroid cells, we studied the following step in the phosphatidylinositol 4,5-diphosphate (PIP_2_) metabolism. External tetrahydrolipstatin (10 µM, a specific inhibitor of DAG lipase) decreased the current magnitude to 10% of the controls (Figure 6C and G), although internal RHC-80267 (another inhibitor of DAG lipase) did not inhibit the current. External methyl arachidonyl fluorophosphonate (MAFP, 1 µM, an inhibitor of MAG lipase and PLA_2_) also decreased the current magnitude to 6% of the controls (Figure 6E and G). After washout of those drugs, the recovery of the response could not be observed. Those results suggest that the DAG lipase-MAG lipase pathway may be involved in the generation of extracellular Ca^2+^-induced current. In the DAG lipase-MAG lipase pathway, DAG is converted to arachidonic acid. External eicosatetraynoic acid (20 µM, ETYA, a non-metabolizable analog of arachidonic acid) did not induce a conductance increase in the cells, but the drug decreased extracellular Ca^2+^-induced current to 5% of the controls (Figure 7A and C). Washout recovered the response to 48% of the controls. It is known that ETYA is a non-specific inhibitor of phospholipase A_2_ (PLA_2_), epoxygenase (cytochrome P-450), cycloxygenase (COX) and lipoxygenase (LO). Baicalein (20 µM, an inhibitor of 12/15-lipoxygenase) decreased the current magnitude to 64% of the controls and the moderate inhibition was significant (Figure 7C). MS-PPOH (20 µM, an inhibitor of epoxygenase) and dicrofenac (20 µM, an inhibitor of cycloxygenase) did not inhibit the extracellular Ca^2+^-induced current (Figure 7C).

### Effects of intracellular arachidonic acid and 12(S)-HPETE

**Figure 8:**
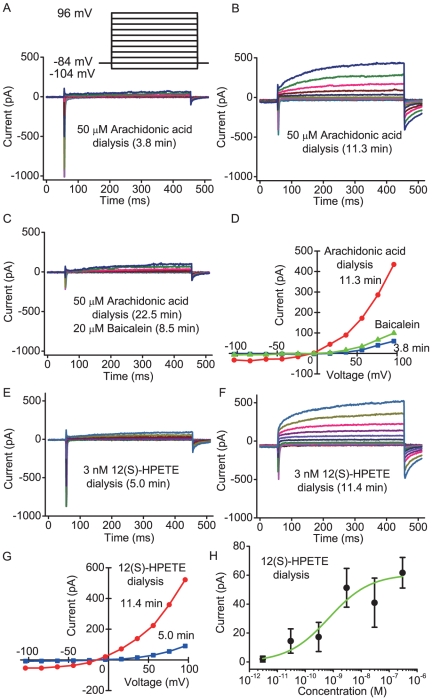
Biophysical properties of intracellular arachidonic acid-induced and 12(S)-HPETE-induced currents. (A) The currents elicited by 400 ms voltage steps between −104 to +96 mV in 20 mV increments from a holding potential of −84 mV under the condition of 50 µM arachidonic acid dialysis after 3.8 min of the membrane rupture in normal saline solution. (B) The currents under same condition after 11.3 min of the rupture. (C) The currents under the condition after 8.5 min of the addition of 20 µM baicalein to external solution. (D) Current/voltage (*I/V*) relationships for the currents at the end of pulse in above conditions. (E) The currents under the condition of 3 nM 12(S)-HPETE dialysis at 5.0 min of the membrane rupture in normal saline solution. (F) The currents under same condition at 11.4 min of the rupture. (G) Current/voltage (*I/V*) relationships for the currents at the end of pulse in above conditions. (H) Dose-response relationship for intracellular 12(S)-HPETE-induced current measured at −54 mV. The ordinate scale denotes the absolute value of the inward current. Data were fitted by the Hill equation (Hill coefficient: 0.6). Values are mean ± from 4–8 cells.

Intracellular dialysis of 50 µM arachidonic acid induced slow developing inward current and the response was decreased by ETYA to 6% of the controls in six cells (Figure 7D and F). Baicalein (20 µM) and niflumic acid (100 µM) also decreased the arachidonic acid-induced currents to 28% and 11% of the controls, respectively (Figure 7F). The reversal potentials of arachidonic acid-induced currents shifted in accordance to the equilibrium potential of Cl^−^ (E_Cl_)(Figure S2). Internal arachidonic acid also elicited slowly activating time-dependent outward currents in response to depolarizing steps and deactivating tail currents on the return to −84 mV from depolarized potentials (Figure 8B). Both components of the currents were greatly inhibited by baicalein (Figure 8C and D, three cells). In steady state during arachidonic acid dialysis, some cells displayed a linear current/voltage (*I/V*) relationships in response to depolarizing steps. Ca^2+^-free saline solution containing 1 mM EGTA gradually decreased the arachidonic acid-induced current to 20% of the control (n = 3), suggesting that the IP_3_-independent Ca^2+^ store might be depleted by sustained action of an arachidonic acid metabolite. Intracellular dialysis of 12 (S)-HPETE at very low concentration also induced time-dependent outward currents and deactivating tail currents (Figure 8F). The apparent EC_50_ for intracellular 12 (S)-HPETE was about 700 pM (Figure 8H). Intracellular dialysis of 1 µM 15(S)-HETE elicited the response (−67±31 pA, n = 5) equivalent to that of 300 nM 12(S)-HPETE (−62±11 pA, n = 6).

### Effects of MAP Kinase Cascade Inhibitor on Extracellular Ca^2+^-induced Current

It has been reported that extracellular Ca^2+^ activates MAP kinase and phospholipase A_2_ via protein tyrosine kinase (PTK) as well as protein kinase C (PKC) in bovine parathyroid cells [9]. When genistein (40 µM, an inhibitor of PTK) was added to the external solution, extracellular Ca^2+^-induced current decreased to 6% of the controls (Figure S3A and E). Washout of the drug recovered the current response to 87% of the controls. External PDBu (10 µM, an activator of PKC) did not evoke the current response, although the drug induced very slowly developing inhibition of voltage-gated Na^+^ current (the time constant of about 10 min) [24]. External PD98059 (50 µM, an inhibitor of MAP kinase) also decreased extracellular Ca^2+^-induced current to 31% of the controls (Figure S3C and E). The current response recovered to 57% of the controls after washout.

## Discussion

The present study shows that frog parathyroid cells can respond to high extracellular Ca^2+^, resulting in an activation of Ca^2+^-activated Cl^−^ conductance. High extracellular Ca^2+^ increased intracellular Ca^2+^ concentration ([Ca^2+^]_i_), but the increase was independent of inositol 1,4,5-trisphosphate (IP_3_). Alternatively, the PLC-DAG-MAG pathway may produce arachidonic acid in frog parathyroid cells. Since ETYA (a non-metabolizable analog of arachidonic acid) could not elicit the current response, arachidonic acid also may not be an intracellular mediator for raising [Ca^2+^]_i_. An arachidonic acid metabolite produced by the action of baicalein-sensitive 12-/15-lipoxygenase may increase [Ca^2+^]_i_. 12-lipoxygenase produces 12(S)-HPETE, while 15-lipoxygenase produces 15(S)-HETE. In the present study, 12(S)-HPETE as well as 15(S)-HETE elicited Ca^2+^-activated Cl^−^ currents. In porcine parathyroid cells, high extracellular Ca^2+^ and arachidonic acid inhibit PTH secretion and baicalein restores the secretion [30]. Both 12(S)-HPETE and 15(S)-HETE inhibit the PTH secretion [31]. No effect of IP_3_ dialysis suggests other targets for the lipid messenger than the endoplasmic reticulum. The intra-granular Ca^2+^ concentration in secretory cells is lower than that in the endoplasmic reticulum but higher than that in the cytosol [32]. 12(S)-HPETE may affect the secretory granules, resulting in the inhibition of PTH secretion. 12(S)-HPETE has been reported to be an extracellular direct ligand for S-K^+^ channels in *Aplysia* [33]. Next, 12(S)-HPETE was confirmed to be a direct activator of TRPV1 [34]. Furthermore, 12-lipoxygenase products induce inflammation in adipocytes [35], reduce insulin secretion from human islets [36] and regulate hippocampal long-term potentiation through the modulation of L-type Ca^2+^ channels [37].

In murine parotid and pancreatic acinar cells, arachidonic acid activates Ca^2+^-selective channels in plasma membrane [38]. The Ca^2+^ entry through the channels increases [Ca^2+^]_i_. It is claimed that the molecular architecture of the arachidonate-regulated Ca^2+^-selective channel is a pentameric assembly of Orai1 and Orai3 subunits [39]. In olfactory transduction, odorant-induced currents in olfactory receptor neurons are amplified by Ca^2+^-activated Cl^−^ current [40]. The transduction currents are divided into cyclic nucleotide-gated cationic and Ca^2+^-activated Cl^−^ conductance. In the present study, Ba^2+^ in Ca^2+^-free saline solution containing 1 mM EGTA also could elicit Ca^2+^-activated Cl^−^ current in frog parathyroid cells. Furthermore, high extracellular Ca^2+^ and intracellular arachidonic acid could induce almost only niflumic acid-sensitive currents. An arachidonic acid metabolite may not induce the Ca^2+^ entry through Ca^2+^-permeable channels in the plasma membrane, but the metabolite may release Ca^2+^ from an unknown novel store independent of IP_3_. This mechanism for [Ca^2+^]_i_ increase without Ca^2+^ entry may make the precise monitoring of [Ca^2+^]_o_ possible. On the other hand, the IP_3_-independent Ca^2+^ store should be refilled by an innate mechanism. In the present experiment, the wash-out of Ca^2+^-free saline solution with normal saline solution for 10 minutes did not recover the arachidonic acid-induced current. Lu et al. [23] identified expression and association of TRPC channels with Orai1 and STIM1 in human parathyroid.

EC_50_ of extracellular Ca^2+^ for inhibition of human PTH secretion is about 1.2 mM [41]. On the other hand, *Xenopus* oocytes injected with cRNA prepared from bovine CaR displayed an EC_50_ of 5 mM extracellular Ca^2+^ for stimulation of Ca^2+^-activated Cl^−^ current under the condition of resting 0.5 mM Ca^2+^ [42]. In the frogs, the actual relationship between extracellular Ca^2+^ concentration and PTH secretion is unknown. Normal saline in the present study contained 1.8 mM Ca^2+^. The higher EC_50_ (6 mM) in this study suggests that the isolated parathyroid cells might fairly desensitize during exposure to 1.8 mM Ca^2+^. Bovine parathyroid cells also displayed the desensitization of the intracellular Ca^2+^ response during sustained extracellular Ca^2+^ stimulation [25]. Nevertheless, we cannot deny that PTH secretion in the frog can be inhibited by extracellular Ca^2+^ between 2 mM and 3 mM.

Ca^2+^-activated Cl^−^ channels carry out the important functions in several tissues including fluid secretion from exocrine gland, amplification of olfactory transduction and block of polyspermy in amphibian oocytes [43]. Recently, the Tmem16 family is recognized to possess characteristics most similar to the native channels [44]–[46]. Similar channels may be expressed in the plasma membrane of frog parathyroid cells. However, the relation between Ca^2+^-activated Cl^−^ channels and inhibition of PTH secretion is unknown. In secretory glands, a low pH of secretory vesicles makes them ready to release their contents. Acidification of secretory granules is carried out by H^+^-ATPase that pumps H^+^ into the vesicular lumen [47]. Lumen-positive voltage across the granular membrane produced by H^+^ fluxes that would prevent further H^+^ pumping can be cancelled with charge compensation. Acidic granules principally depend on Cl^−^ fluxes for charge neutralization. In accordance with this mechanism, procedures reducing intracellular Cl^−^ concentration decrease influx of Cl^−^ through granular Cl^−^ channels, resulting in inhibition of exocytosis [48]. In the present study, we tried the prevention of the Cl^−^ permeation through the patch membrane using gramicidin instead of amphotericin B to estimate natural intracellular Cl^−^ concentration. The reversal potentials of extracellular Ca^2+^-induced currents shifted from −25 mV in early stage (1 min) to −58 mV in steady state (3 min). This suggests that initial concentration of intracellular Cl^−^ was 51 mM, and that the concentration decreased to 14 mM in a few minutes. We suppose that the decrease of intracellular Cl^−^ concentration in parathyroid cells also may inhibit influx of Cl^−^ through granular Cl^−^ channels. The inhibition may lead to the reduction in priming of PTH secretory granules for release, which is constructed by Cl^−^-dependent proton pumping.

Kifor et al. [9] reported that high extracellular Ca^2+^ regulates MAP kinase through the mediation protein kinase C (PKC) and protein tyrosine kinase (PTK) in bovine parathyroid cells, resulting in PLA_2_ activation. In the present study, genistein greatly decreased extracellular Ca^2+^-induced current. Genistein is an isoflavone that interacts with several molecules in living cells. Genistein affects voltage-gated Na^+^ current in the neurons through PTK-dependent and kinase-independent mechanisms [49]. Similarly, genistein greatly decreased voltage-gated Na^+^ current in frog parathyroid cells to 2% of the controls (data not shown). Alternatively, genistein also may affect PLCγ, because PLCγ has Src homology, which has tyrosine kinase activity [50]. Further study using a specific inhibitor for PTK should be performed in order to elucidate PTK-dependent mechanisms.

In conclusion, frog parathyroid cells use a novel mechanism for extracellular Ca^2+^-sensing.

## Materials and Methods

### Cell preparation

Adult bullfrogs (*Rana catesbeiana*) weighing 250–550 g were used for the experiment over the course of a year. The experiments were performed in accordance with the Guidelines for Animal Experimentation of Nagasaki University with approval of the Institutional Animal Care and Use Committee. The keeping of bullfrogs (invasive alien species) was approved by the Ministry of the Environment of Japan (approval number 06000204). Parathyroid cells were isolated from the parathyroid glands of decapitated and pithed animals. Two pairs of the oval parathyroid glands in both sides were quickly dissected from the precordial region which lie near the ventral branchial bodies and attached to the carotid arteries [51]. The glands were cut into small pieces in Ca^2+^-free saline containing 2 mM EDTA and incubated for 10–12 min in 2 ml of same saline containing 10 mM L-cysteine and 10 units/ml papain (Sigma, St. Louis, MO, USA). The glands were then rinsed with normal saline. The individual cells were dissociated by gentle trituration in normal saline. Isolated parathyroid cells displayed an oval shape, with a diameter of about 10 µm [24].

### Electrophysiological recording

Voltage-clamp recording was performed in whole-cell configuration [52] using a CEZ 2300 patch-clamp amplifier (Nihon Kohden, Tokyo, Japan) or an EPC-7 plus amplifier (HEKA Elektronik, Lambrecht, Germany). The patch pipettes were pulled from Pyrex glass capillaries containing a fine filament (Summit Medical, Tokyo, Japan) with a two-stage puller (Narishige PD-5, Tokyo, Japan). The tips of the electrodes were heat-polished with a microforge (Narishige MF-80). The resistance of the resulting patch electrode was 5–10 MΩ when filled with internal solution. The formation of 5–20 GΩ seals between the patch pipette and the cell surface was facilitated by applying weak suction to the interior of the pipette. The patch membrane was broken by applying strong suction, resulting in a sudden increase in capacitance. Amphotericin B (133–160 µg/ml, Sigma) or gramicidin (100 µg/ml, Sigma) was added to the pipette solution when using the perforated method [53]. The perforated whole-cell condition was obtained within 5 min of the establishment of a GΩ seal. Recordings were made from parathyroid cells that had been allowed to settle on the bottom of a chamber placed on the stage of an inverted microscope (Olympus IMT-2, Tokyo, Japan). The recording pipette was positioned with a hydraulic micromanipulator (Narishige WR-88). The current signal was low-pass-filled at 5 kHz, digitized at 125 kHz using a TL-1 interface (Axon Instruments, Union City, CA, USA), acquired at a sampling rate of 0.25–5 kHz using a computer running the pCLAMP 5.5 software (Axon Instruments), and stored on a hard disk. The pCLAMP was also used to control the digital-analogue converter for the generation of the clamp protocol. The indifferent electrode was a chlorided silver wire. The voltages were corrected for the liquid junction potential between external solution and internal solution. Capacitance and series resistance were compensated for, as appropriate. The whole-cell current-voltage (*I/V*) relationship was obtained from the current generated by the 167 mV/s voltage ramp from −100 to +100 mV. In some case, the current-voltage relationship was obtained from the current generated the 400 ms voltage-step pulses between −104 and +96 mV in 20 mV increments from a holding potential of −84 mV. Input resistance was calculated from the slope conductance generated by the voltage ramp from −104 to −54 mV. Data were analyzed with pCLAMP and Origin 7.5 and 8.0 (Origin Lab, Northampton, MA, USA). Unless stated otherwise, the data are presented as means ± S.E.M., significance was tested by Student's t test and a difference was considered significant if P<0.05.

### Measurement of [Ca^2+^]_i_ using fura-2

Dissociated parathyroid cells in normal saline solutions collected into the recording chamber were incubated at room temperature in 10 µM fura-2 acetoxymethyl ester (fura-2 AM, Dojindo Laboratories, Kumamoto, Japan) with 0.02% cremophore EL (Nacalai, Kyoto, Japan) and washed thoroughly with normal saline after 30 min. Cell adhesive, concavalin A (type IV, Sigma) was coated to the bottom of the recording chamber to immobilize the cells during the experiment. A chamber containing fura-2-loaded cells was placed on the stage of an inverted microscope (Diaphot 300, Nikon, Tokyo, Japan). A 100-W xenon lamp emitted the excitation light. The fluorescence was imaged using a silicon-intensified target (SIT) camera (C2400-08, Hamamatsu Photonics, Hamamatsu, Japan) and digitized to 8 bits per pixel with an Argus 50 image processor system (Ratio imaging program, version 3.5, Hamamatsu). A PC controlled the filter and intensifier gains. Imaging data were stored on magneto-optical disks. Changes in fluorescence ratios of excitation at 340 nm and 380 nm light pulses were measured in the selected areas to determine [Ca^2+^]_i_ responses. We used a light-attenuating filter for 340 nm excitation light to balance the brightness of the two images. Measurements were taken only during first 2 h after completion of loading the fura-2 due to the reduction in fura-2 level. Measurement of fluorescence ratios under a continuous perfusion with normal saline solution was carried out for 5 min to confirm the stable basal level before applying any chemical. Switching between solutions was electronically controlled.

### Solution and drugs

Normal saline solution consisted of (in mM): NaCl, 115; KCl, 2.5; CaCl_2_, 1.8; Hepes, 10; glucose, 20; pH 7.2. The pH of normal saline and other solution was adjusted by Tris base. The external Na^+^-free 10 mM Ba^2+^ solution was prepared by the replacement of Na^+^,K^+^ and Ca^2+^ with NMDG^+^ and Ba^2+^. The solution exchange was done by gravity flow. For stock solution, spermine tetrahydrochloride (100 mM, Sigma) and ruthenium red (10 mM, Sigma) were dissolved in deionized water. WIN 55,212-2 mesylate (10 mM, Tocris, Bristol, UK), phorbol 12,13-dibutyrate (PDBu, 10 mM, Sigma), U73122 (5 mM, Tocris and Sigma), U73343 (2 mM, Calbiochem, San Diego, USA), ryanodine (20 mM, Sigma), 1-oleoyl-2-acetyl-*sn*-glycerol (OAG, 50 mM, Sigma), arachidonic acid (50 mM, Sigma and Cayman, Ann Arbor USA), RHC-80267 (10 mM, Calbiochem), tetrahydrolipstatin (10 mM, Sigma), 5,8,11,14-eicosatetraynoic acid (ETYA, 20 mM, Sigma), baicalein (50 mM, Cayman), MS-PPOH (10 mM, Cayman), dicrofenac (50 mM, Cayman), genistein (20 mM, Sigma), PD98059 (50 mM, Cayman) and m-3M3FBS (50 mM, Tocris) were dissolved in dimethylsulphoxide (DMSO). 2-arachidonoylglycerol (2-AG, 26.4 mM) dissolved in acetonitrile, 2-arachidonoylglycerol ether (2-AG ether, 13.7 mM), 12S-hydroperoxy-5Z,8Z,10E,14Z-eicosatetraenoic acid (12(S)-HPETE, 297 µM) and 15S-hydroxy-5Z,8Z,11Z,13E-eicosateraenoic acid (15(S)-HETE, 312 µM) dissolved in ethanol and methyl arachidonyl fluorophosphonate (MAFP, 27 mM) dissolved in methyl acetate were purchased from Cayman. Samples of the stock solutions were added to normal saline solution or internal solution to give the desired final concentrations. NPS R-467 (100 µM, NPS Pharmaceuticals, Salt Lake City, USA), NPS S-467 (100 µM, NPS Pharmaceuticals), and niflumic acid (100–300 µM, Sigma) were directly dissolved in normal saline solution. The standard K^+^ internal solution contained (in mM): KCl, 100; CaCl_2_, 0.1; MgCl_2_, 2; EGTA, 1; Hepes, 10; pH 7.2. In some experiments, internal K^+^ was replaced with Cs^+^ and Cl^−^ was replaced with gluconate^−^. BAPTA (10 mM, Sigma), GTP (1 mM, Sigma), GDPβS (1 mM, Sigma), 1,4,5-IP_3_ (50 µM, Sigma) and cyclic AMP (1 mM, Sigma) were dissolved in internal solution.

All experiments were carried out at room temperature (20–25°C).

## Supporting Information

Figure S1 **Extracellular Ba^2+^-induced current in frog parathyroid cells.** (A)The plot of the whole-cell current/voltage (*I/V*) relationship produced by a voltage ramp. Extracellular cations (Na^+^, K^+^ and Ca^2+^) other than Ba^2+^ were replaced with NMDG^+^. (B) The shift of the reversal potential of extracellular Ba^2+^-induced current in another cell. Intracellular Cl^−^ concentration was reduced from 104 to 10 mM. (C) Extracellular Ba^2+^-induced current in the condition of external Ca^2+^ elimination with EGTA. (D) The *I/V* relationship of the difference between a and b in the pen recording C. (E) Comparison of extracellular Ba^2+^-induced currents measured at −54 mV with and without extracellular Ca^2+^. The pipette solution contained amphotericin B (133 µg/ml).
(EPS)

Figure S2 **Effects of baicalein and niflumic acid on intracellular arachidonic acid-induced currents.** (A)The plot of the whole-cell currents/voltage (*I/V*) relationships produced by a voltage ramp of internal arachidonic acid-induced currents under the condition of high intracellular Cl^−^ concentration (E_Cl_ = −4 mV) and the inhibition with 20 µM baicalein. (B) The similar relationships under the condition of low intracellular Cl^−^ (E_Cl_ = −63 mV). (C) Inhibition of the internal arachidonic acid-induced currents with 100 µM niflumic acid.
(EPS)

Figure S3 **Effects of genistein and PD98059 on extracellular Ca^2+^-induced current.** (A) The pen recording of the inhibitory effect of external genistein on extracellular Ca^2+^-induced current at −54 mV. (B) The plots of the whole-cell *I/V* relationships during control, genistein application and wash-out. The relationships were obtained from the pen recording on the upper part. (C) The pen recording of the inhibitory effect of external PD98059 on extracellular Ca^2+^-induced current at −54 mV. (D) The plots of the whole-cell *I/V* relationships during control, PD98059 application and wash-out. The relationships were obtained from the pen recording on the upper part. (E) Comparison of the effects of genistein and PD98059 on extracellular Ca^2+^-induced current at −54 mV. The pipette solution contained amphotericin B (133 µg/ml).
(EPS)
